# Supplementary material for: Pressure-induced ferroelectric-like transition creates a polar metal in defect antiperovskites Hg3Te2X2 (X = Cl, Br)
Source: Nat Commun. 2021 Mar 8;12:1509. doi: 10.1038/s41467-021-21836-7 (PMC7940478; doi:10.1038/s41467-021-21836-7)
Supplement: Supplementary file 1 — Supplementary Information [file 41467_2021_21836_MOESM1_ESM.pdf]

Supplementary Information

Pressure-induced ferroelectric-like transition creates a polar metal in defect antiperovskites $Hg_3Te_2X_2$ (X = Cl, Br)

Weizhao Cai[1], Jiangang He[2*], Hao Li[3], Rong Zhang[1], Dongzhou Zhang[4], Duck Young Chung[3], Tushar Bhowmick[1], Christopher Wolverton[2], Mercouri G. Kanatzidis[3,5*], and Shanti Deemyad[1*]

[1]Department of Physics and Astronomy, University of Utah, Salt Lake City, Utah 84112, United States

[2]Department of Materials Science and Engineering, Northwestern University, Evanston, Illinois 60208, United States

[3]Materials Science Division, Argonne National Laboratory, Lemont, Illinois 60439, United States

[4]PX2, Hawaii Institute of Geophysics and Planetology, University of Hawaii at Manoa, Honolulu, Hawaii 96822, United States

[5]Department of Chemistry, Northwestern University, Evanston, Illinois 60208, United States

*To whom correspondence should be addressed.

J.H. (email: jiangang2020@gmail.com)

M.G.K. (email: m-kanatzidis@northwestern.edu)

S.D. (email: Deemyad@physics.utah.edu)

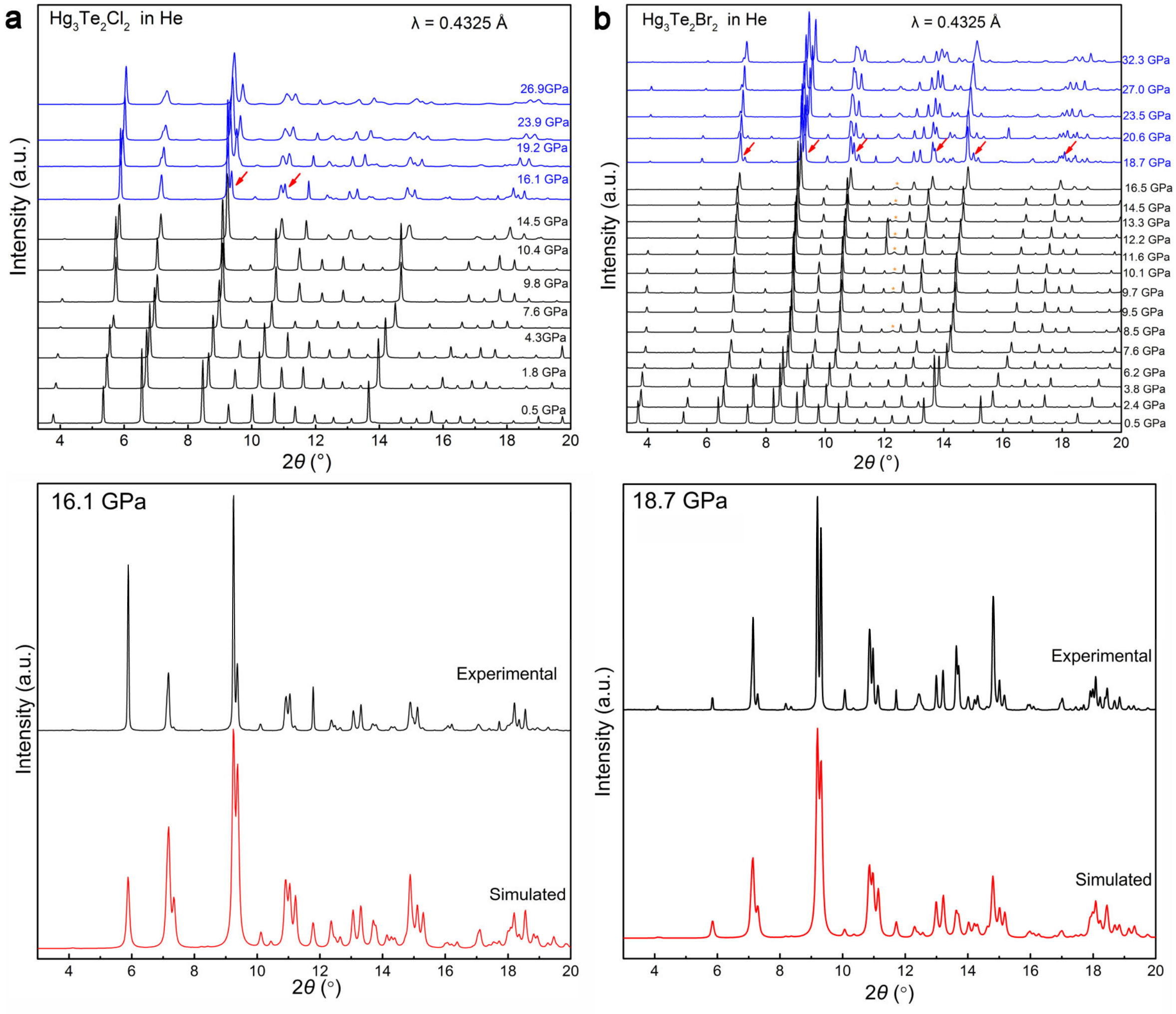

Supplementary Fig. 1. High pressure single crystal X-ray diffraction patterns of $Hg_3Te_2X_2$ (X = Cl, Br). (**a**) $Hg_3Te_2Cl_2$ and (**b**) $Hg_3Te_2Br_2$ collected from wide image scan using He as the PTM at room temperature. Phases I and II are indicated as black and blue, respectively. The bottom panels show the comparison of the experimental X-ray diffraction patters and simulated patterns of phase II from solved single crystal structures at 16.1 GPa for $Hg_3Te_2Cl_2$ and 18.7 GPa for $Hg_3Te_2Br_2$, respectively.

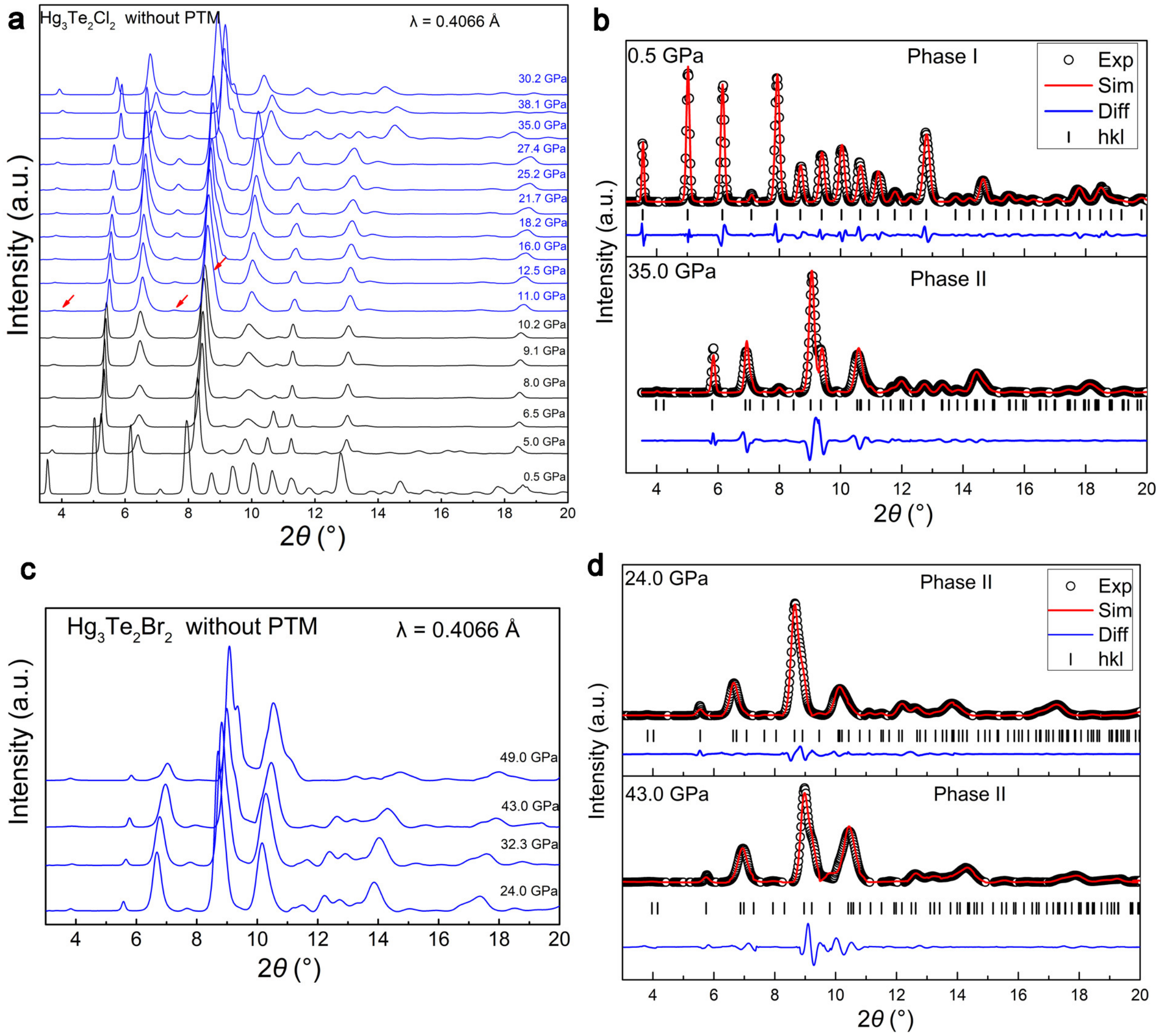

Supplementary Fig. 2. Synchrotron X-ray diffraction patterns of $Hg_3Te_2X_2$ (X = Cl, Br) at different pressures. (**a**) Powder X-ray diffraction patterns of $Hg_3Te_2Cl_2$ sample without pressure medium compressed to 38.1 GPa at room temperature. (**b**) Le Bail fit of X-ray data at 0.5 and 35.0 GPa. (**c**) X-ray diffraction patterns of $Hg_3Te_2Br_2$ sample without pressure medium compressed from 24.0 to 49 GPa at room temperature. (**d**) Le Bail fits of the data at 24.0 and 43.0 GPa. The black circles are the measured scattering intensity, and the red solid line represents the fit to the data. The vertical bars indicate Bragg reflection positions of the phase I and II together with difference profiles (blue lines) shown at the bottom.

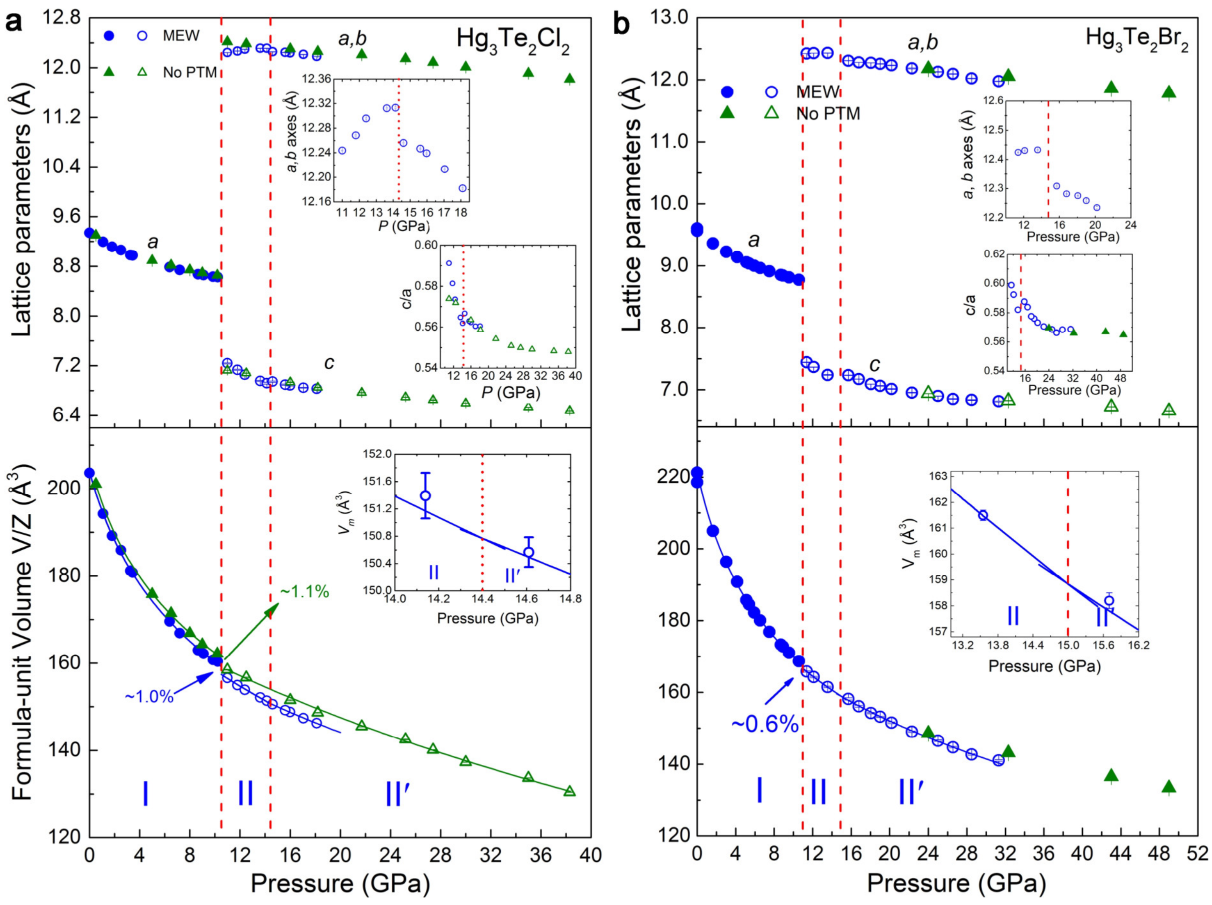

Supplementary Fig. 3. Lattice parameters of $Hg_3Te_2X_2$ (X = Cl, Br) compressed under different hydrostatic conditions at room temperature. (**a**) $Hg_3Te_2Cl_2$ and (**b**) $Hg_3Te_2Br_2$. The insets in the upper panels of (**a**) and (**b**) show pressure-dependent lattice parameters *a* and *b* and the *c*/*a* ratio of phase II. The second- and third-order Birch-Murnaghan equation of state fit to the formula-unit volume (*V*/*Z*) data. These equations of state have been used for calculating the ΔV drop at transition between phases I/II and II/II′. The inset enhances the second-order phase transition of phase II-II′ in MEW. The calculated bulk moduli are given in Supplementary Table 1. Vertical red dashed lines indicate two phase transitions at 10.5 and 14.4 GPa for $Hg_3Te_2Cl_2$, 11.0 and 15.0 GPa for $Hg_3Te_2Br_2$ compressed in the MEW. In addition, the phase I-II transition in $Hg_3Te_2Cl_2$ without pressure medium also shown in red lined at ~10.5 GPa in (**a**).

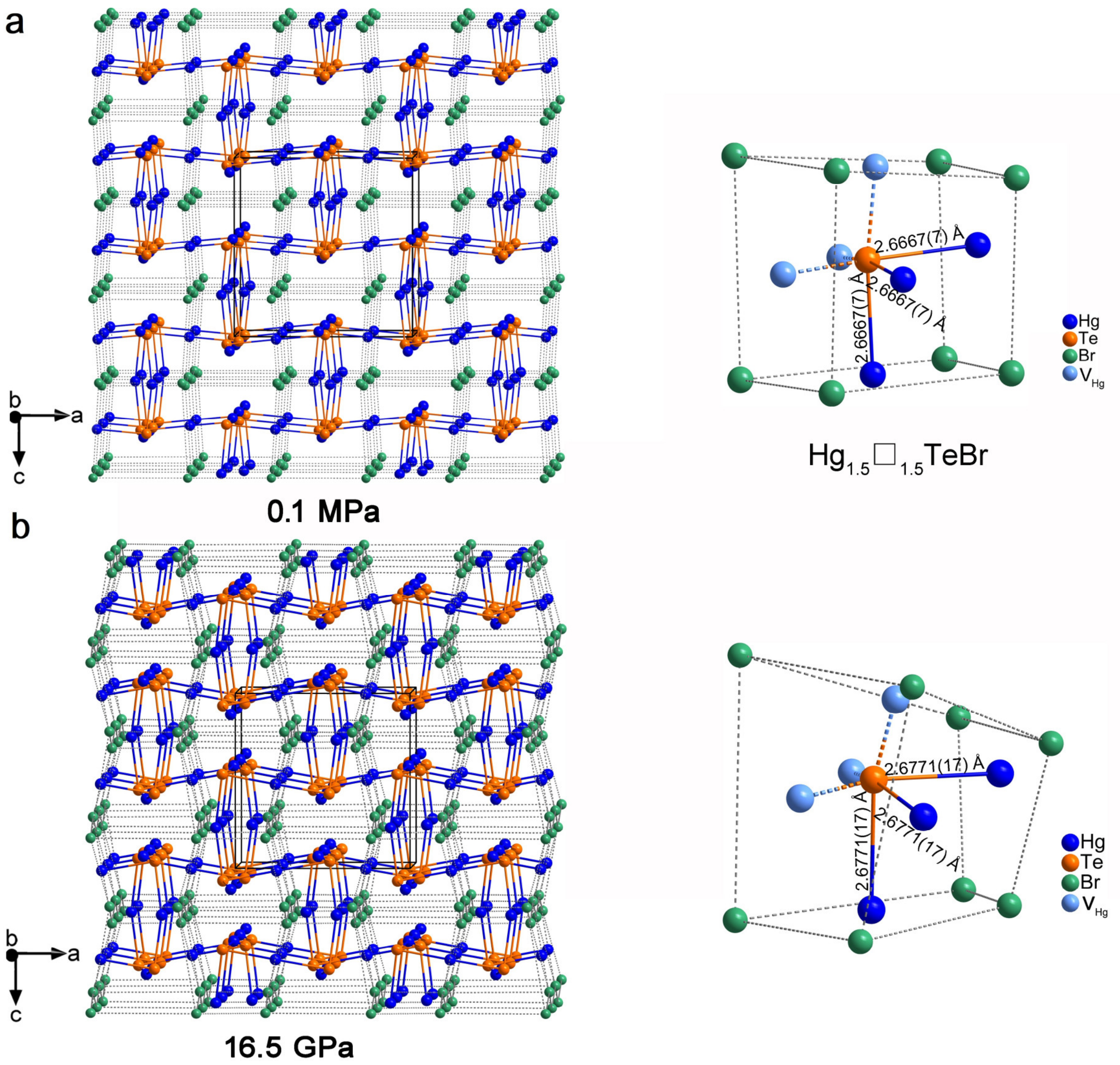

Supplementary Fig. 4. Structures of phase I-He of $Hg_3Te_2Br_2$ at different pressures. (**a**) 0.1 MPa. (**b**) 16.5 GPa viewed approximately along the [010] direction. The structural evolution of defect antiperovskite structure of $Hg_{1.5}\square_{1.5}TeBr$ under compression.

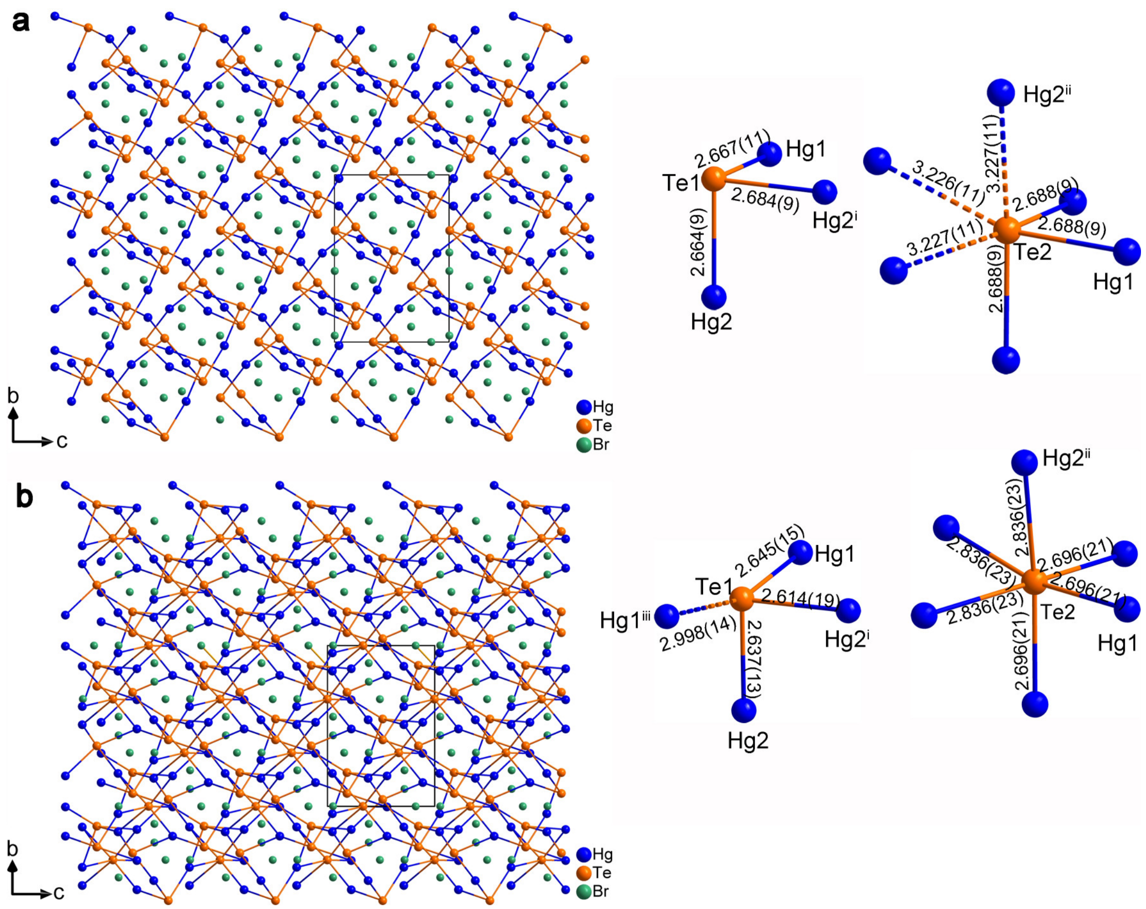

Supplementary Fig. 5. Comparison of structures of II-He of $Hg_3Te_2Br_2$ at different pressures. (**a**) 18.7 and (**b**) 32.3 GPa. Coordination numbers of Te1 and Te2 atoms. Symmetry codes: (i) 1/3–*x*+*y*, 2/3–*x*, *z*–1/3; (ii) *x*, *y*, *z*–1; (iii) 2/3–*x*+*y*, 1/3–*x*, *z*–2/3.

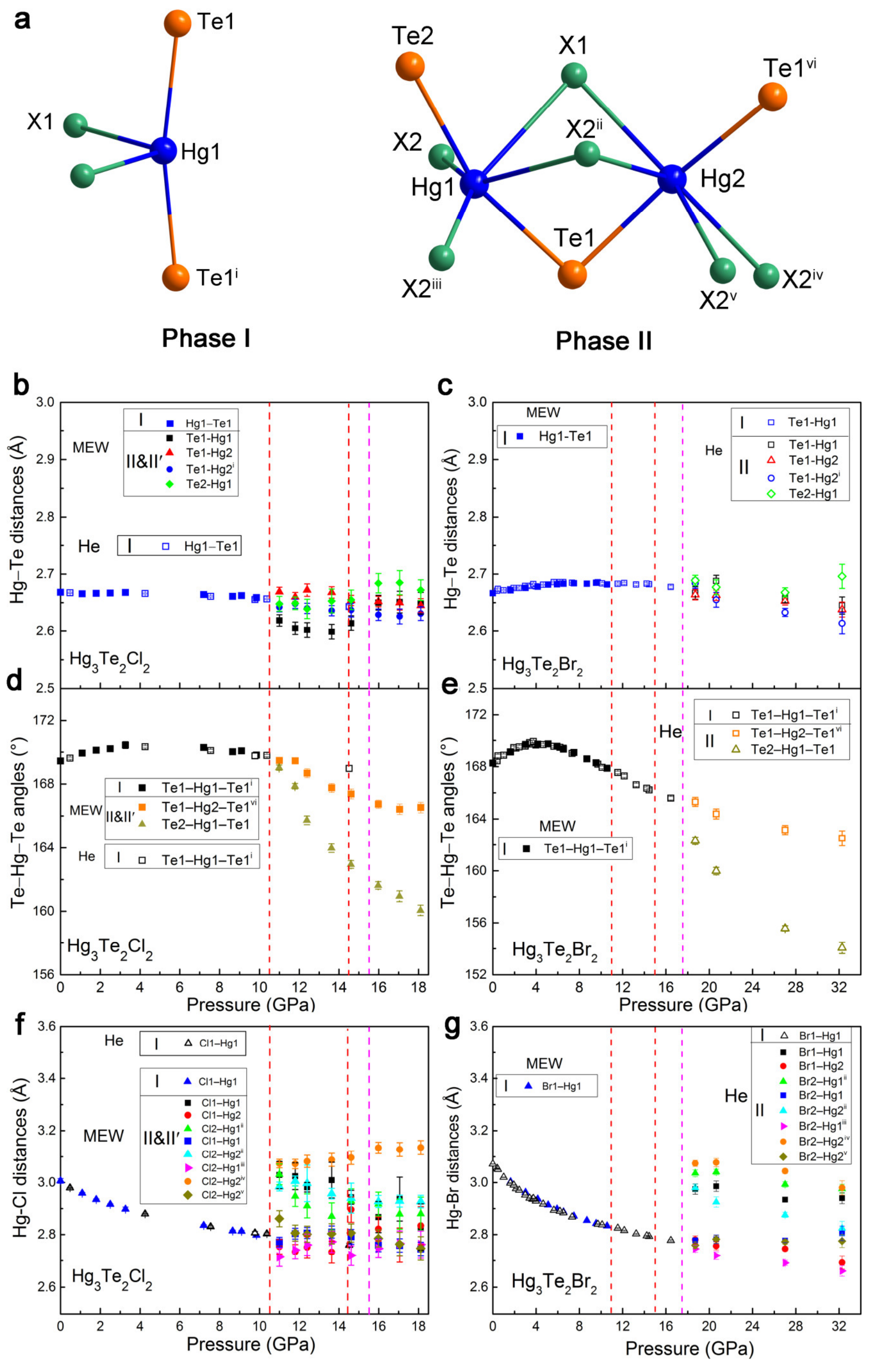

Supplementary Fig. 6. Evolution of bond lengths and angles of $Hg_3Te_2X_2$ (X = Cl, Br) as a function of pressure. (**a**) The coordination environment of Hg atoms in phases I and II′. Evolution of Hg–Te distances as a function of pressure of (**b**) $Hg_3Te_2Cl_2$ and (**c**) $Hg_3Te_2Br_2$. Pressure dependence of Te–Hg–Te angles in (**d**) $Hg_3Te_2Cl_2$ and (**e**) $Hg_3Te_2Br_2$. The Hg–X distances as a function of pressure in (**f**) $Hg_3Te_2Cl_2$ and (**g**) $Hg_3Te_2Br_2$. Symmetry codes in phase I: (i) 2–*y*, *z*–0.5, 1.5–*x*. In phases II: (i) 1/3–*x*+*y*, 2/3–*x*, *z*–1/3; (ii) *x*, *y*, *z*–1; (iii) 1–*y*, 1+ *x*–*y*, *z*; (iv) 2/3–*x*+*y*, 4/3–*x*, *z*+1/3; (v) 1/3+*x*, 2/3+*y*, *z*–2/3; (vi) 2/3–*x*+*y*, 4/3–*x*, *z*–2/3.

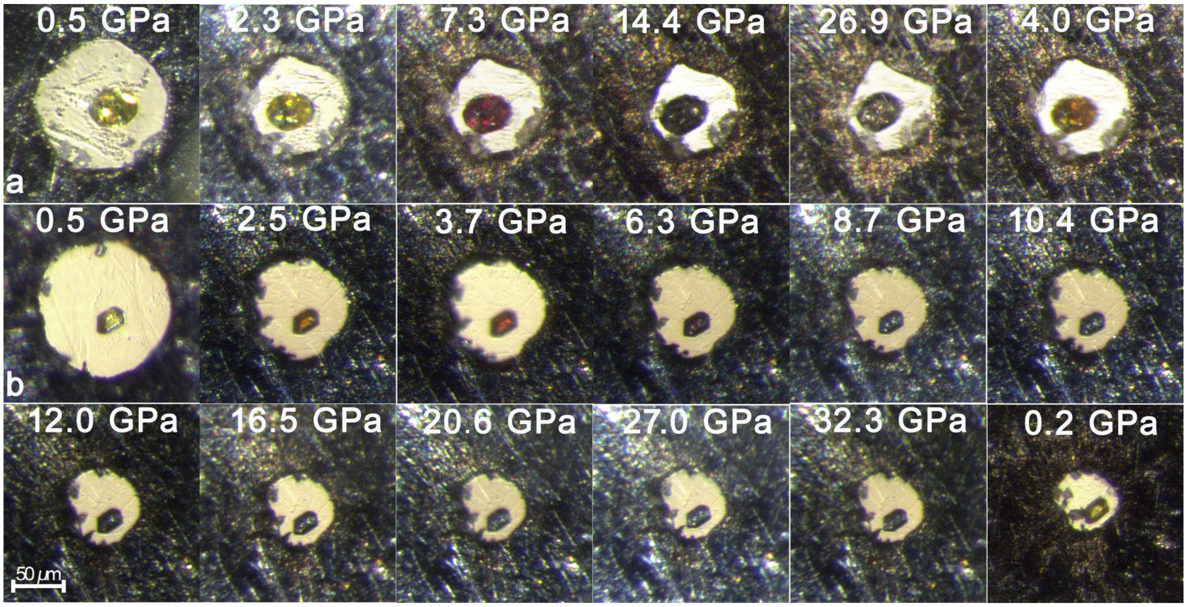

Supplementary Fig. 7. Optical micrographs of $Hg_3Te_2X_2$ (X = Cl, Br) single crystals under pressure. (**a**) $Hg_3Te_2Cl_2$ and (**b**) $Hg_3Te_2Br_2$ single crystals compressed in He to 26.9 and 32.3 GPa, respectively. The crystal color was completely recovered when pressure released to low pressure. Note that the sample chambers shrink under compression due to the softness of He.

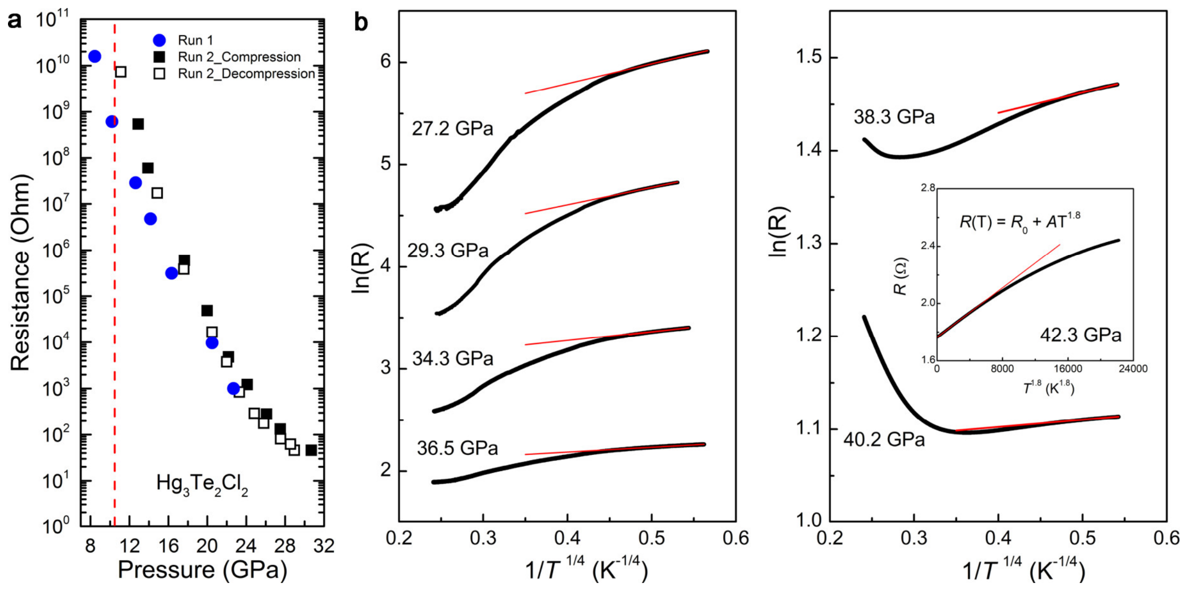

Supplementary Fig. 8. Pressure-dependent electrical resistances of $Hg_3Te_2X_2$ (X = Cl, Br). (a) Pressure-dependent electrical resistances of $Hg_3Te_2Cl_2$ at room temperature. The filled and unfilled squares indicate the data measured from compression and decompression, respectively. (**b**) 3-D VRH model $\ln R$-$1/T^{1/4}$ at 27.2, 29.3, 34.3, 36.5, 38.3 and 40.2 GPa of $Hg_3Te_2Br_2$. The inset shows $T^{1.8}$ dependence of the resistance at pressure of 42.3 GPa (below 70 K), which is consistent with the formula $R(T) = R_0 + AT^{1.8}$ with $R_0 = 1.76\ \Omega$ and A = 4.32×10^{-5}.

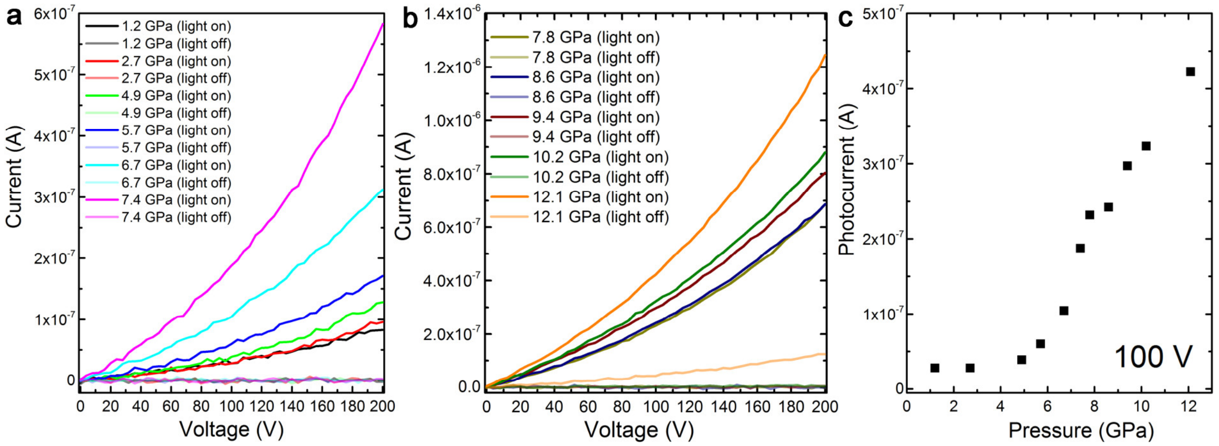

Supplementary Fig. 9. (Photo)current of $Hg_3Te_2Br_2$ as function of voltage under pressures at room temperature. (**a**) 1.2 to 7.4 GPa, (**b**) 7.8 to 12.1 GPa. (**c**) Changes of photocurrent as an evolution of pressure at 100 V.

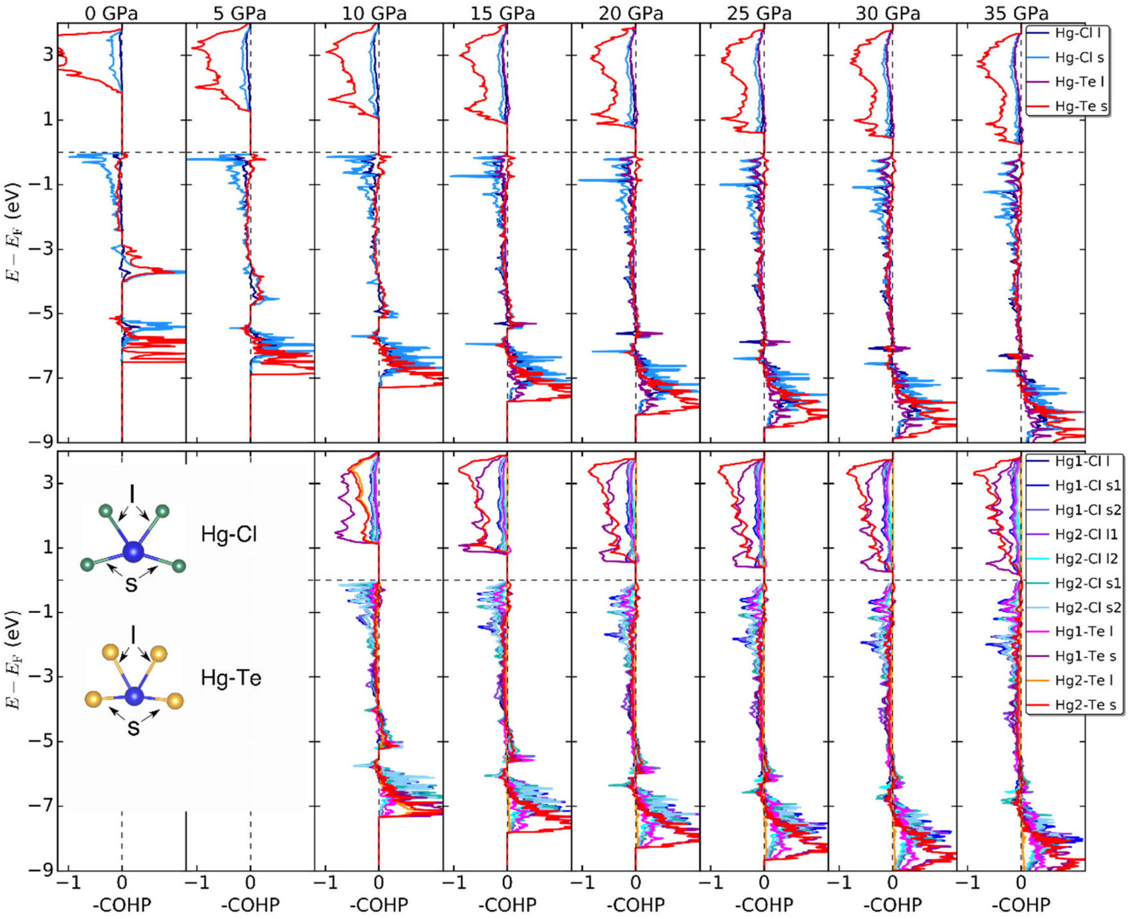

Supplementary Fig. 10. Crystal orbital Hamilton Population (COHP). Evolution of -COHP as a function of pressure for $I2_13$ (upper planer) and $R3$ (lower planer) phases. The l and s are the average bond lengths of two long and short Hg–Cl and Hg–Te bonds, which are defined in lower left corner of the figure.

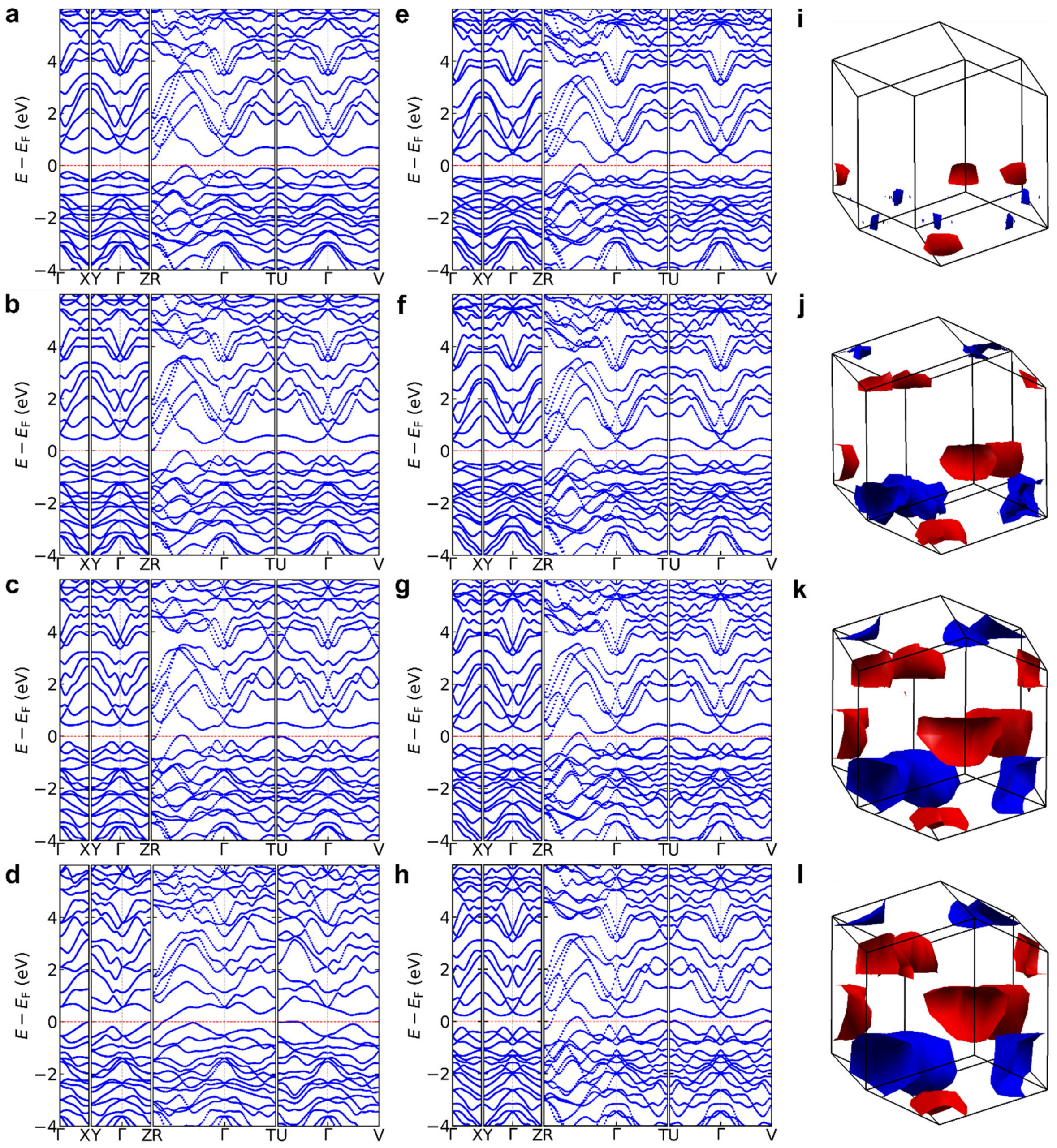

Supplementary Fig. 11. PBEsol electronic band structures of *R*3 phase of $Hg_3Te_2X_2$ under different pressures. (**a**), (**b**), (**c**) and (**d**) are band structures of $Hg_3Te_2Cl_2$ at 30, 35, 40, and 45 GPa, respectively. (**e**), (**f**), (**g**) and (**h**) are band structures of $Hg_3Te_2Br_2$ at 30, 35, 40, and 45 GPa, respectively. (**i**), (**j**), (**k**) and (**l**) are the Fermi surfaces of $Hg_3Te_2Br_2$ at 30, 35, 40, and 45 GPa, respectively. Red and blue colors represent valance and conduction bands, respectively.

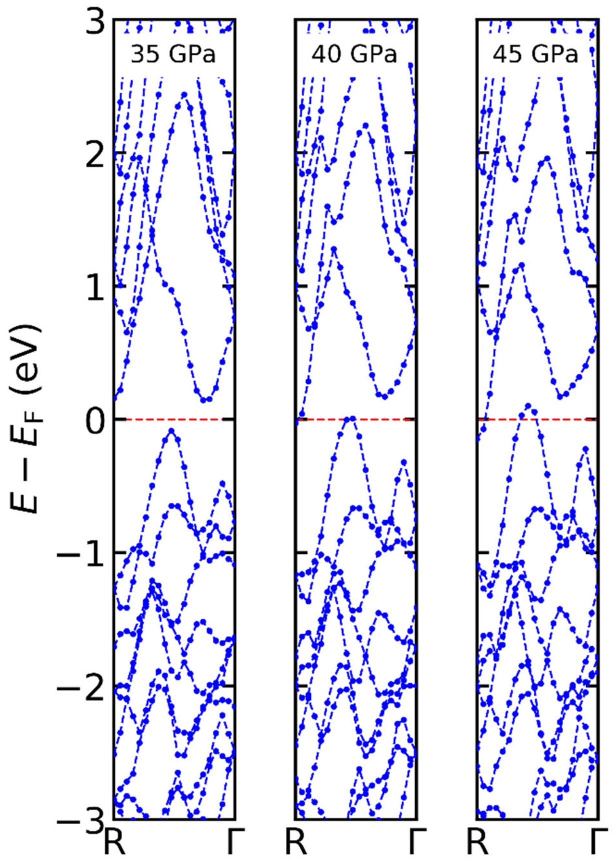

Supplementary Fig. 12. Evolution of electronic band structures of *R*3 phase of $Hg_3Te_2Br_2$ under pressure calculated by using HSE06. Only the band structure along the high symmetry points R and Γ is computed due to the heavy cost of HSE06 calculation. With the pressure increasing from 35 GPa to 45 GPa, $Hg_3Te_2Br_2$ becomes semimetal and metal gradually.

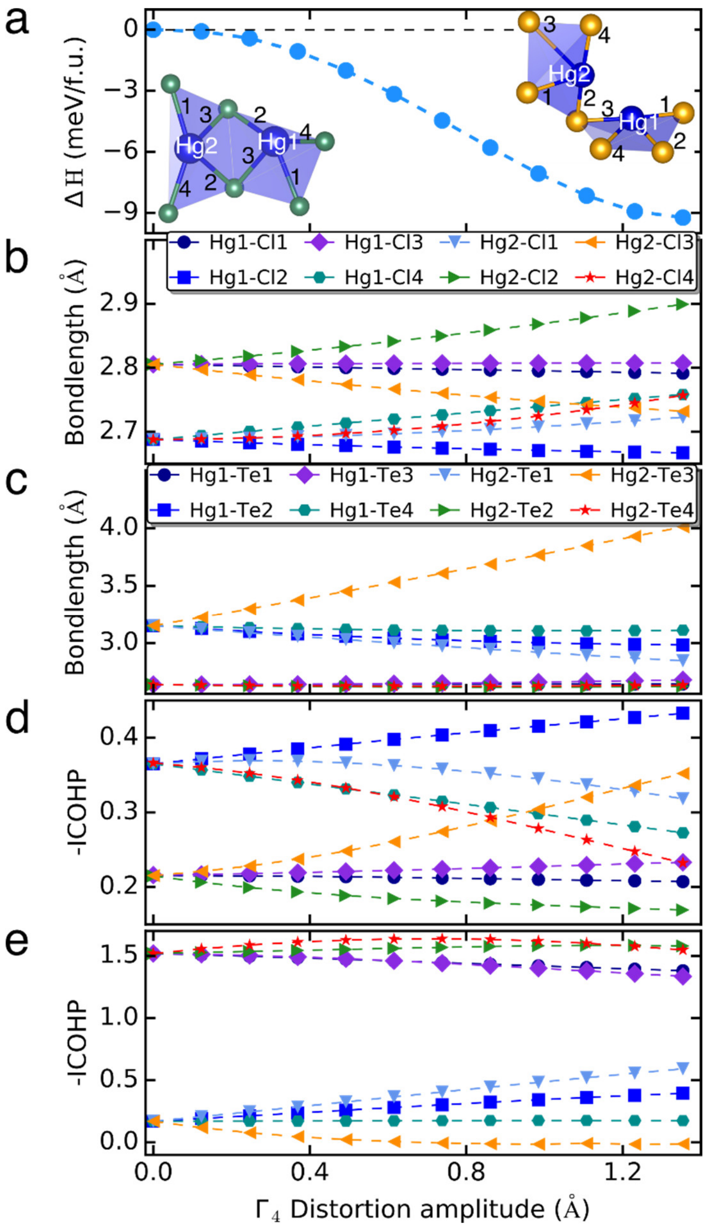

Supplementary Fig. 13. Structure and bonding evolution of $Hg_3Te_2Cl_2$ as a function of Γ_4 distortion at 30 GPa. (**a**) Enthalpy difference (ΔH) with respect to $I2_13$ phase. (**b**) bond length of Hg–Cl bonds. (**c**) bond length of Hg–Te bonds. (**d**) -ICOHP of Hg–Cl pairs. (**e**) -ICOHP of Hg–Te pairs.

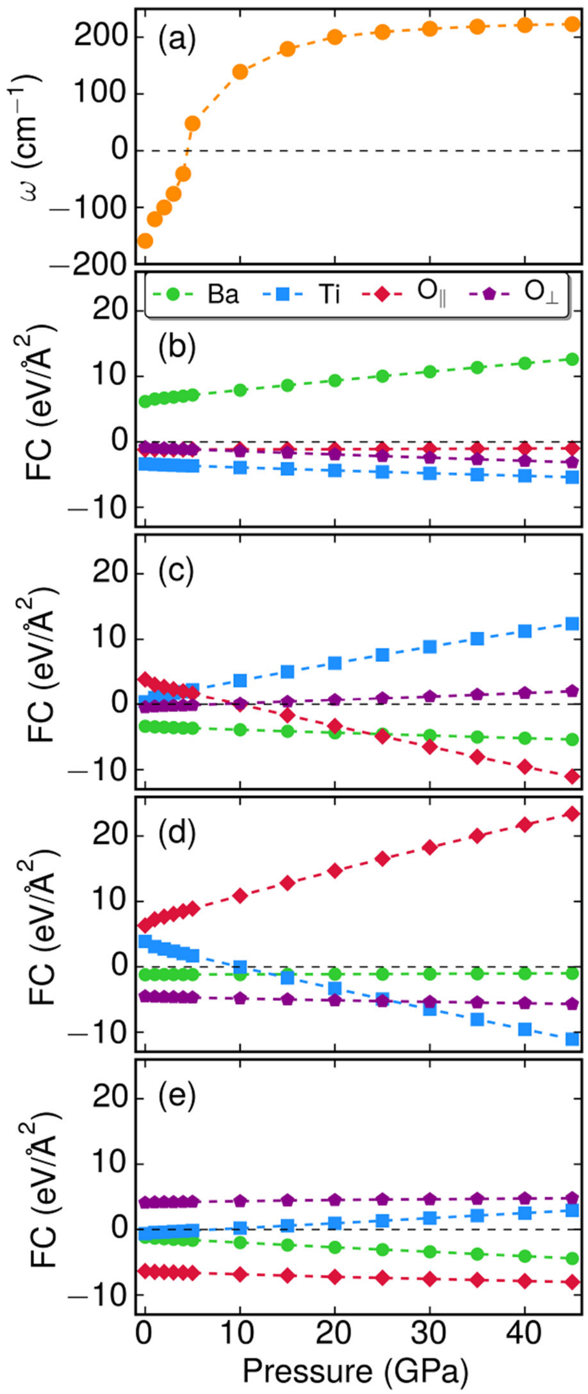

Supplementary Fig. 14. Pressure dependence of frequency and force constants of $BaTiO_3$. (a) Frequency of ferroelectric mode Γ_4^- of cubic $BaTiO_3$. (**b**) Force constants of cubic $BaTiO_3$ with the Ba atom displacing along [001] direction. (**c**) Force constants of cubic $BaTiO_3$ with the Ti atom displacing along [001] direction. (**d**) Force constants of cubic $BaTiO_3$ with the out-of-plane oxygen ($O_\parallel$) displacing along [001] direction. (**e**) Force constants of cubic $BaTiO_3$ with the in-plane oxygen ($O_\perp$) displacing along [001] direction.

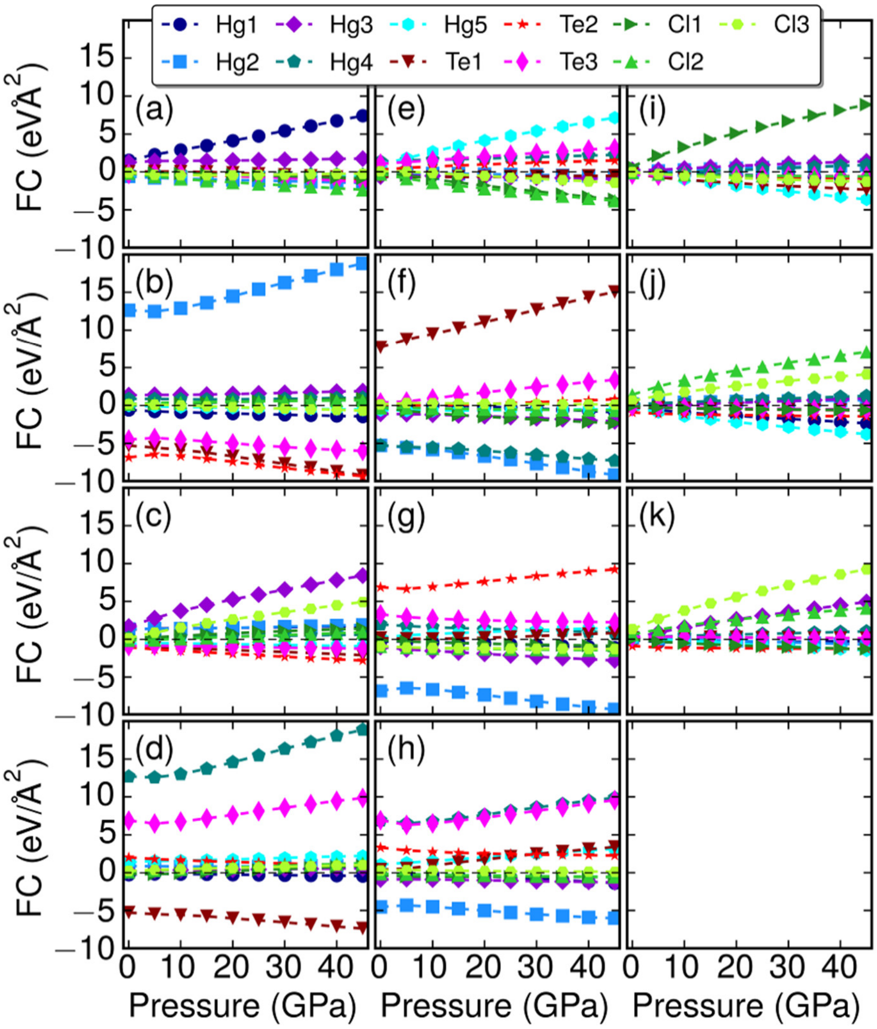

Supplementary Fig. 15. Γ$_4$ phonon modes force constants of phase *I*2$_1$3 of $Hg_3Te_2Cl_2$ under pressure. (**a-k**) Force constants of symmetry equivalent atom groups when one atom group is displaced. The atom groups generated by Smodes are listed below. The largest FCs at each subplot is the on-site FCs.

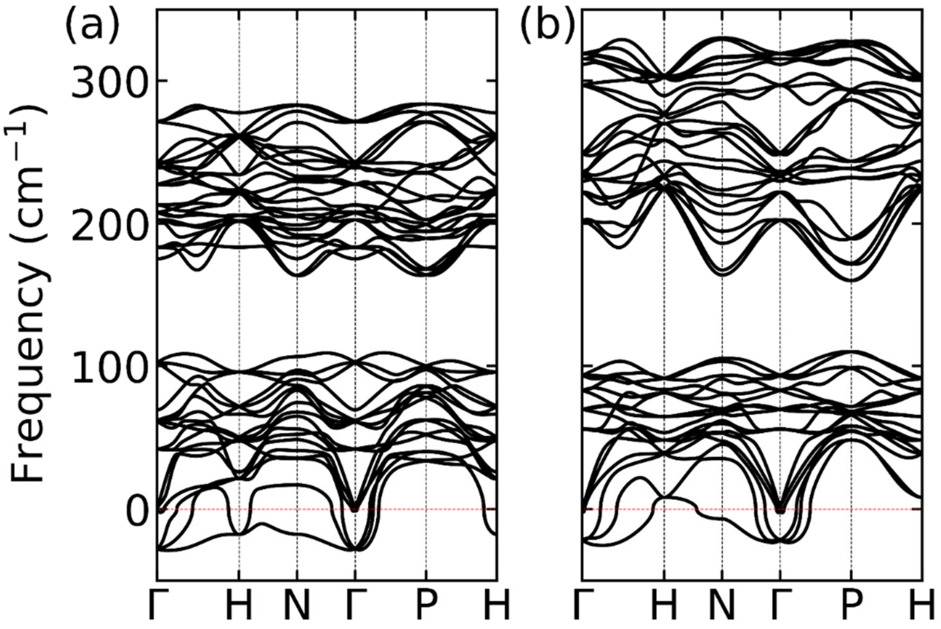

Supplementary Fig. 16. Phonon dispersion of *I*2$_1$3 phase of (a) $Hg_3Se_2F_2$ and (b) $Hg_3S_2Cl_2$ at 20 GPa.

Supplementary Table 1. Calculated bulk moduli of $Hg_3Te_2X_2$ samples under different hydrostatic conditions

$Hg_3Te_2Cl_2$				
Hydrostatic media	Pressure range (GPa)	Equations of state fitted	B_0 (GPa)	B'
MEW	0–10.2 (phase I)	3rd-order BM EOS	18.8(17)	6.7(29)
	11.0–14.2 (phase II)	2nd-order BM EOS	46(5)	4
	14.6–18.1 (phase II′)	2nd-order BM EOS	61(7)	4
He	0.5–14.5 (phase I)	3rd-order BM EOS	17.4(21)	7.5(8)
	16.1–25.9 (phase II)	2nd-order BM EOS	63.3(19)	4
Without medium	0.5–10.2 GPa (Phase I)	3rd-order BM EOS	17.4(26)	7.7(12)
	11.0–38.3 GPa (Phase II)	3rd-order BM EOS	88(8)	2.7(3)
$Hg_3Te_2Br_2$				
MEW	0–10.6 (I-MEW)	3rd-order BM EOS	17.3(9)	5.9(3)
	11.4–13.6 (II-MEW)	2nd-order BM EOS	38(3)	4
	15.7–31.3 (III-MEW)	2nd-order BM EOS	51(3)	4
He	0–16.5 GPa (I-He)	3rd-order BM EOS	19.4(2)	5.5(1)
	18.7–32.3 (II-He)	2nd-order BM EOS	52.1(17)	4

Supplementary Table 2. Lattice parameters of $Hg_3Te_2Cl_2$ at variable pressures compressed in He and MEW

P (GPa)	a (Å)	b (Å)	c (Å)	V (Å^3)	Phase
Pressure medium: He					
0.5	9.2686(2)	9.2686(2)	9.2686(2)	796.24(3)	**I-He**
1.8	9.1258(3)	9.1258(3)	9.1258(3)	760.01(6)	
4.3	8.9247(2)	8.9247(2)	8.9247(2)	710.85(3)	
7.6	8.7368(1)	8.7368(1)	8.7368(1)	666.89(13)	
9.8	8.6381(2)	8.6381(2)	8.6381(2)	644.55(3)	
10.4	8.6157(2)	8.6157(2)	8.6157(2)	639.55(3)	
14.5	8.4869(4)	8.4869(4)	8.4869(4)	611.29(5)	
16.1	12.0560(5)	12.0560(5)	7.1314(15)	897.7(2)	**II-He**
18.5	12.0638(5)	12.0638(5)	6.9968(5)	881.87(8)	
23.9	11.9743(7)	11.9743(7)	6.8806(7)	854.4(1)	
26.9	11.9139(8)	11.9139(8)	6.8099(8)	837.1(1)	
Pressure medium: MEW					
1.1	9.1933(3)	9.1933(3)	9.1933(3)	776.99(4)	**I-MEW**
1.8	9.1130(2)	9.1130(2)	9.1130(2)	756.81(3)	
2.5	9.0601(3)	9.0601(3)	9.0601(3)	743.70(4)	
3.3	8.9831(4)	8.9831(4)	8.9831(4)	724.90(6)	
7.2	8.7399(2)	8.7399(2)	8.7399(2)	667.60(3)	
8.7	8.6705(2)	8.6705(2)	8.6705(2)	651.83(3)	
9.1	8.6567(2)	8.6567(2)	8.6567(2)	648.72(3)	
9.9	8.6315(2)	8.6315(2)	8.6315(2)	643.07(3)	
11.0	12.2436(7)	12.2436(7)	7.237(7)	939.5(10)	**II-MEW**
11.8	12.2682(9)	12.2682(9)	7.137(9)	930.3(12)	
12.4	12.2957(10)	12.2957(10)	7.052(10)	923.3(13)	
13.6	12.3123(10)	12.3123(10)	6.950(9)	912.4(12)	
14.1	12.3131(13)	12.3131(13)	6.918(15)	908(2)	
14.6	12.2559(12)	12.2559(12)	6.935(10)	902.1(13)	**II'-MEW**
16.0	12.2390(8)	12.2390(8)	6.882(7)	892.8(9)	
17.1	12.2133(9)	12.2133(9)	6.844(8)	884.1(10)	
18.1	12.1819(9)	12.1819(9)	6.828(7)	877.5(9)	
15.6§	12.2466(10)	12.2466(10)	6.894(10)	895.4(13)	
10.2§	8.6245(3)	8.6245(3)	8.6245(3)	641.51(4)	**I-MEW**
6.4§	8.7859(3)	8.7859(3)	8.7859(3)	678.21(4)	
3.5§	8.9760(3)	8.9760(3)	8.9760(3)	723.18(4)	
0.0001§	9.3388(3)	9.3388(3)	9.3388(3)	814.47(5)	

§ Decompression data.

Supplementary Table 2 Continued

P (GPa)	a (Å)	b (Å)	c (Å)	V (Å^3)	Phase
Pressure medium: without PTM					
0.5	9.3003(4)	9.3003(4)	9.3003(4)	804.4(1)	**I**
5.0	8.8931(14)	8.8931(14)	8.8931(14)	703.3(4)	
6.5	8.8183(8)	8.8183(8)	8.8183(8)	685.7(2)	
8.0	8.7392(11)	8.7392(11)	8.7392(11)	667.5(3)	
9.1	8.6943(14)	8.6943(14)	8.6943(14)	657.2(4)	
10.2	8.6561(15)	8.6561(15)	8.6561(15)	648.6(4)	
11.0	12.415(2)	12.415(2)	7.124(3)	950.9(4)	**II**
12.5	12.383(2)	12.383(2)	7.081(4)	940.2(6)	
16.0	12.305(3)	12.305(3)	6.933(4)	909.1(7)	
18.2	12.262(2)	12.262(2)	6.849(4)	891.9(5)	
21.7	12.206 (3)	12.206 (3)	6.765(2)	872.9(4)	
25.2	12.146(3)	12.146(3)	6.692(4)	855.0(6)	
27.4	12.086(3)	12.086(3)	6.646(3)	840.6(5)	
35.0	11.910(2)	11.910(2)	6.530(2)	802.1(3)	
38.1	11.815(2)	11.815(2)	6.473(3)	782.5(4)	
30.2[§]	12.010(2)	12.010(2)	6.595(2)	823.8(3)	

[§] Decompression data.

Supplementary Table 3. Unit-cell dimensions of $Hg_3Te_2Br_2$ at variable pressures compressed in MEW and He at room temperature

P (GPa)	*a* (Å)	*b* (Å)	*c* (Å)	*V* (Å^3)	Phase
Pressure medium: He					
0.0001	9.5621(11)	9.5621(11)	9.5621(11)	874.299(11)	Run 1
0.3	9.5206(2)	9.5206(2)	9.5206(2)	862.96(3)	
1.0	9.4174(3)	9.4174(3)	9.4174(3)	835.21(5)	
1.6	9.3515(3)	9.3515(3)	9.3515(3)	817.79(6)	
2.2	9.2778(3)	9.2778(3)	9.2778(3)	798.61(4)	
3.0	9.1981(4)	9.1981(4)	9.1981(4)	778.21(6)	
4.0	9.1193(4)	9.1193(4)	9.1193(4)	758.38(6)	**I-He**
4.7	9.0751(3)	9.0751(3)	9.0751(3)	747.40(6)	
5.9	8.9939(3)	8.9939(3)	8.9939(3)	727.51(4)	
6.5	8.9561(4)	8.9561(4)	8.9561(4)	718.38(6)	
7.5	8.8964(3)	8.8964(3)	8.8964(3)	704.11(4)	
3.8§	9.1370(4)	9.1370(4)	9.1370(4)	762.80(4)	
3.5§	9.1570(5)	9.1570(5)	9.1570(5)	767.82(4)	
0.5	9.5031(1)	9.5031(1)	9.5031(1)	858.22(2)	Run 2
1.9	9.3192(2)	9.3192(2)	9.3192(2)	809.35(3)	
2.4	9.2551(1)	9.2551(1)	9.2551(1)	792.76(2)	
3.8	9.1486(1)	9.1486(1)	9.1486(1)	765.71(1)	
6.2	8.9761(1)	8.9761(1)	8.9761(1)	723.21(1)	
7.6	8.8960(1)	8.8960(1)	8.8960(1)	704.02(4)	
8.5	8.8500(4)	8.8500(4)	8.8500(4)	693.15(10)	
9.5	8.8026(6)	8.8026(6)	8.8026(6)	682.08(10)	
9.7	8.7959(1)	8.7959(1)	8.7959(1)	680.52(1)	**I-He**
10.1	8.7778(2)	8.7778(2)	8.7778(2)	676.33(3)	
11.6	8.7255(1)	8.7255(1)	8.7255(1)	664.31(1)	
12.2	8.6987(1)	8.6987(1)	8.6987(1)	658.21(1)	
13.3	8.6492(2)	8.6492(2)	8.6492(2)	647.04(3)	
14.5	8.6033(2)	8.6033(2)	8.6033(2)	636.79(3)	
14.2	8.6132(2)	8.6132(2)	8.6132(2)	638.99(3)	
16.5	8.5475(2)	8.5475(2)	8.5475(2)	624.48(3)	
18.7	12.1185(5)	12.1185(5)	7.1875(10)	914.09(5)	Run 2
20.6	12.1031(5)	12.1031(5)	7.0876(12)	899.1(2)	
23.5	12.0457(8)	12.0457(8	7.0212(7)	882.25(6)	**II-He**
27.0	11.9653(5)	11.9653(5)	6.9161(11)	857.5(2)	
32.3	11.848(1)	11.848(1)	6.8340(21)	830.8(3)	

Supplementary Table 3 continued

P (GPa)	*a* (Å)	*b* (Å)	*c* (Å)	*V* (Å^3)	Phase
Pressure medium: MEW					
1.7	9.3598(2)	9.3598(2)	9.3598(2)	819.97(3)	**I-MEW**
3.0	9.2268(2)	9.2268(2)	9.2268(2)	785.51(3)	
4.2	9.1386(2)	9.1386(2)	9.1386(2)	763.20(3)	
5.1	9.0568(2)	9.0568(2)	9.0568(2)	742.89(3)	
5.4	9.0367(2)	9.0367(2)	9.0367(2)	737.95(6)	
5.9	9.0001(2)	9.0001(2)	9.0001(2)	729.02(3)	
6.5	8.9638(2)	8.9638(2)	8.9638(2)	720.24(3)	
7.5	8.9107(2)	8.9107(2)	8.9107(2)	707.51(3)	
8.7	8.8508(2)	8.8508(2)	8.8508(2)	693.34(3)	
8.9	8.8420(14)	8.8420(14)	8.8420(14)	691.3(2)	
9.6	8.8111(2)	8.8111(2)	8.8111(2)	684.05(3)	
10.6	8.7709(2)	8.7709(2)	8.7709(2)	674.73(3)	
11.4	12.4249(4)	12.4249(4)	7.4421(17)	994.9(10)	**II-MEW**
12.1	12.4308(11)	12.4308(11)	7.365(2)	985.6(12)	
13.6	12.4331(14)	12.4331(14)	7.238(4)	968.9(12)	
15.7	12.309(2)	12.309(2)	7.234(3)	949.2(13)	**II'-MEW**
19.0	12.259(3)	12.259(3)	7.062(6)	919.1(10)	
20.2	12.2354(4	12.2354(4	7.014(6)	909.3(13)	
22.3	12.1862(5)	12.1862(5)	6.954(5)	894.3(12)	
25.0	12.1309(3)	12.1309(3)	6.899(2)	879.2(12)	
26.6	12.0962(4)	12.0962(4)	6.852(5)	868.2(12)	
28.5	12.0249(4)	12.0249(4)	6.836(5)	855.6(12)	
31.3	11.9764(3)	11.9764(3)	6.813(8)	846.1(14)	
18.1§	12.2757(5)	12.2757(5)	7.901(1)	925.4(12)	**II-MEW**
16.8§	12.2823(8)	12.2823(8)	7.171(2)	936.8(14)	
5.4§	9.0367(2)	9.0367(2)	9.0367(2)	737.95(6)	**I-MEW**
0.0001§	9.6011(3)	9.6011(3)	9.6011(3)	885.05(7)	
Pressure medium: without PTM					
24.0	12.080(2)	12.080(2)	6.938(2)	891.4(3)	**II**
32.3	12.054(3)	12.054(3)	6.823(2)	858.7(5)	
43.1	12.860(3)	12.860(3)	6.724(3)	818.9(5)	
49.0	11.782(4)	11.782(4)	6.657(4)	800.3(7)	

§ Decompression data.

Supplementary Table 4. Selected crystallographic parameters of $Hg_3Te_2Cl_2$ single crystal compressed in helium at room temperature

Pressure (GPa)	0.5	4.3	7.6	10.4	14.5	16.1
CCDC number	2021970	2021971	2021972	2021973	2021974	2021975
Phase	**I-He**					**II-He**
Formula	$Hg_3Te_2Cl_2$					
Pressure medium	He					
Crystal system	Cubic					Rhombohedral
Space group	$I2_13$					$R3$
Crystal size (mm^3)	0.051 × 0.035 × 0.025					
a/Å	9.2686(2)	8.9247(2)	8.7368(1)	8.6157(2)	8.4869(4)	12.0560(5)
b/Å	9.2686(2)	8.9247(2)	8.7368(1)	8.6157(2)	8.4869(4)	12.0560(5)
c/Å	9.2686(2)	8.9247(2)	8.7368(1)	8.6157(2)	8.4869(4)	7.1314(15)
α/°	90	90	90	90	90	90
β/°	90	90	90	90	90	90
γ/°	90	90	90	90	90	120
V/Å^3	796.24(3)	710.85(3)	666.895(13)	639.55(3)	611.29(5)	897.7(2)
Z	4					6
D_{cal} (g/cm^3)	7.740	8.670	9.241	9.637	10.082	10.299
R_{int}	0.1001	0.0895	0.0937	0.0914	0.0751	0.0799
Index ranges	$-7 \le h \le 6$<br>$-14 \le k \le 8$<br>$-10 \le l \le 11$	$14 \le h \le 8$<br>$-6 \le k \le 7$<br>$-10 \le l \le 10$	$-8 \le h \le 14$<br>$-6 \le k \le 7$<br>$-10 \le l \le 10$	$-5 \le h \le 7$<br>$-8 \le k \le 14$<br>$-10 \le l \le 10$	$-4 \le h \le 6$<br>$-13 \le k \le 8$<br>$-9 \le l \le 10$	$-11 \le h \le 17$<br>$-15 \le k \le 11$<br>$-5 \le l \le 7$
R_1/wR_2 [$I>2\sigma(I)$][a]	0.0480/0.1198	0.0443/0.1253	0.0464/0.1210	0.0465/0.1206	0.0635/0.1478	0.1141/0.2905
R_1/wR_2 (all data)	0.0503/0.1202	0.0504/0.1273	0.0501/0.1218	0.0514/0.1227	0.0649/0.1485	0.1205/0.2920
Goodness of fit on F^2	1.064	1.099	1.047	1.066	1.263	1.131
Largest peak/hole (e·Å^{-3})	2.36/–2.51	4.00/–2.33	4.62/–3.05	5.40/–2.80	5.78/–4.92	5.67/–4.46

[a] $R_1=\sum||F_o|-|F_c||/\sum|F_o|$ for $F_o^2>2\sigma(F_o^2)$; $wR_2=\sum[w(F_o^2-F_c^2)]/\sum[w(F_o^2)^2]^{1/2}$,where $w=1/[\sigma^2F_o^2+(A\,P)^2+BP]$, and $P=(Fo^2+2Fc^2)/3$

Supplementary Table 5. Selected crystallographic parameters of $Hg_3Te_2Br_2$ single crystal compressed in helium at room temperature

Pressure (GPa)	0.5	2.4	6.2	9.7	11.6	13.3	16.5
CCDC number	2021956	2021957	2021958	2021959	2021960	2021961	2021962
Phase	**I-He**						
Formula	$Hg_3Te_2Br_2$						
Pressure medium	He						
Crystal system	Cubic						
Space group	$I2_13$						
Crystal size (mm^3)	0.030 × 0.020 × 0.010						
a/Å	9.5031(1)	9.2551(1)	8.9761(1)	8.7959(1)	8.7255(1)	8.6492(2)	8.5475(2)
b/Å	9.5031(1)	9.2551(1)	8.9761(1)	8.7959(1)	8.7255(1)	8.6492(2)	8.5475(2)
c/Å	9.5031(1)	9.2551(1)	8.9761(1)	8.7959(1)	8.7255(1)	8.6492(2)	8.5475(2)
α/°	90	90	90	90	90	90	90
β/°	90	90	90	90	90	90	90
γ/°	90	90	90	90	90	90	90
V/Å^3	858.215(16)	792.763(15)	723.208(14)	680.520(13)	664.310(13)	647.04(3)	624.48(3)
Z	4						
D_{cal} (g/cm^3)	7.869	8.519	9.339	9.924	10.166	10.438	10.815
R_{int}	0.0725	0.0693	0.0914	0.0909	0.0791	0.0673	0.0729
Index ranges	$-6 \le h \le 9$<br>$-8 \le k \le 15$<br>$-9 \le l \le 7$	$-6 \le h \le 9$<br>$-8 \le k \le 15$<br>$-9 \le l \le 7$	$-6 \le h \le 9$<br>$-8 \le k \le 14$<br>$-9 \le l \le 6$	$-7 \le h \le 9$<br>$-14 \le k \le 7$<br>$-8 \le l \le 6$	$-5 \le h \le 8$<br>$-14 \le k \le 7$<br>$-7 \le l \le 9$	$-8 \le h \le 5$<br>$-7 \le k \le 14$<br>$-7 \le l \le 9$	$-5 \le h \le 8$<br>$-7 \le k \le 14$<br>$-9 \le l \le 6$
R_1/wR_2 [$I>2\sigma(I)$][a]	0.0442/0.0986	0.0477/0.1226	0.0505/0.1290	0.0534/0.1364	0.0465/0.1240	0.0514/0.1329	0.0425/0.1062
R_1/wR_2 (all data)	0.0452/0.0986	0.0483/0.1228	0.0516/0.1292	0.0553/0.1364	0.0484/0.1242	0.0514/0.1329	0.0429/0.1063
Goodness of fit on F^2	1.173	1.229	1.091	1.166	1.169	1.121	1.128
Largest peak/hole (e·Å^{-3})	2.19/–1.17	2.33/–2.33	3.32/–3.31	4.22/–2.18	3.95/–2.42	4.06/–3.43	3.62/–2.48

[a] $R_1=\sum||F_o|-|F_c||/\sum|F_o|$ for $F_o^2>2\sigma(F_o^2)$; $wR_2=\sum[w(F_o^2-F_c^2)]/\sum[w(F_o^2)^2]^{1/2}$,where $w=1/[\sigma^2F_o^2+(A\,P)^2+BP]$, and $P=(Fo^2+2Fc^2)/3$

Supplementary Table 5 Continued

Pressure (GPa)	18.7	20.6	27.0	32.3
CCDC number	2021963	2021964	2021965	2021966
Phase	**II-He**			
Formula	$Hg_3Te_2Br_2$			
Pressure medium	He			
Crystal system	Rhombohedral			
Space group	*R*3			
Crystal size (mm^3)	0.030 × 0.020 × 0.010			
a/Å	12.1185(5)	12.1031(5)	11.9653(5)	11.8480(10)
b/Å	12.1185(5)	12.1031(5)	11.9653(5)	11.8480(10)
c/Å	7.1875(1)	7.0876(12)	6.9161(11)	6.834(2)
α/°	90	90	90	90
β/°	90	90	90	90
γ/°	120	120	120	120
V/Å^3	914.13(5)	899.13(16)	857.5(2)	830.8(3)
Z	6			
D_{cal} (g/cm^3)	11.082	11.267	11.814	12.194
R_{int}	0.0780	0.0693	0.0511	0.0691
Index ranges	$12 \le h \le 17$<br>$-17 \le k \le 11$<br>$-5 \le l \le 7$	$-14 \le h \le 11$<br>$-11 \le k \le 14$<br>$-7 \le l \le 4$	$-13 \le h \le 13$<br>$-12 \le k \le 17$<br>$-4 \le l \le 7$	$-12 \le h \le 12$<br>$-15 \le k \le 11$<br>$-6 \le l \le 4$
R_1/wR_2 [I>2σ(I)][a]	0.0914/0.2270	0.0664/0.1827	0.0526/0.1428	0.0956/0.2386
R_1/wR_2 (all data)	0.0946/0.2272	0.0685/0.1827	0.0557/0.1430	0.0977/0.2387
Goodness of fit on F^2	1.096	1.187	1.108	1.141
Largest peak/hole (e·Å^{-3})	4.18/–4.10	4.48/–4.30	4.72/–4.27	5.47/–4.57

[a] $R_1=\sum||F_o|-|F_c||/\sum|F_o|$ for $F_o^2>2\sigma(F_o^2)$; $wR_2=\sum[w(F_o^2-F_c^2)]/\sum[w(F_o^2)^2]^{1/2}$,where $w=1/[\sigma^2F_o^2+(A\ P)^2+BP]$, and $P=(Fo^2+2Fc^2)/3$

Atom groups generated by Smodes.

Lattice vectors:

-4.66592	4.66592	4.66592
4.66592	-4.66592	4.66592
4.66592	4.66592	-4.66592

atom type position

1	Hg	2.97714	0.00000	2.33296
2	Hg	1.68878	0.00000	6.99888
3	Hg	0.00000	2.33296	2.97714
4	Hg	0.00000	6.99888	1.68878
5	Hg	2.33296	2.97714	0.00000
6	Hg	6.99888	1.68878	0.00000
7	Te	2.65426	2.65426	2.65426
8	Te	-2.01166	2.01166	6.67758
9	Te	6.67758	-2.01166	2.01166
10	Te	2.01166	6.67758	-2.01166
11	Cl	0.15799	0.15799	0.15799
12	Cl	0.15799	-0.15799	4.50793
13	Cl	4.50793	0.15799	-0.15799
14	Cl	-0.15799	4.50793	0.15799

Hg1 group

1	Hg	1.00000	0.00000	0.00000
2	Hg	1.00000	0.00000	0.00000
3	Hg	0.00000	0.00000	1.00000
4	Hg	0.00000	0.00000	1.00000
5	Hg	0.00000	1.00000	0.00000

6	Hg	0.00000	1.00000	0.00000

Hg2 group

1	Hg	0.00000	1.00000	0.00000
2	Hg	0.00000	1.00000	0.00000
3	Hg	1.00000	0.00000	0.00000
4	Hg	1.00000	0.00000	0.00000
5	Hg	0.00000	0.00000	1.00000
6	Hg	0.00000	0.00000	1.00000

Hg3 group

1	Hg	0.00000	0.00000	1.00000
2	Hg	0.00000	0.00000	-1.00000
3	Hg	0.00000	1.00000	0.00000
4	Hg	0.00000	-1.00000	0.00000
5	Hg	1.00000	0.00000	0.00000
6	Hg	-1.00000	0.00000	0.00000

Hg4 group

1	Hg	0.00000	1.00000	0.00000
2	Hg	0.00000	-1.00000	0.00000
3	Hg	1.00000	0.00000	0.00000
4	Hg	-1.00000	0.00000	0.00000
5	Hg	0.00000	0.00000	1.00000
6	Hg	0.00000	0.00000	-1.00000

Hg5 group

1	Hg	0.00000	0.00000	1.00000
2	Hg	0.00000	0.00000	1.00000
3	Hg	0.00000	1.00000	0.00000
4	Hg	0.00000	1.00000	0.00000
5	Hg	1.00000	0.00000	0.00000
6	Hg	1.00000	0.00000	0.00000

Te1 group

7	Te	1.00000	1.00000	1.00000
8	Te	-0.33333	0.33333	0.33333
9	Te	0.33333	-0.33333	0.33333
10	Te	0.33333	0.33333	-0.33333

Te2 group

8	Te	1.00000	0.50000	0.50000
9	Te	0.50000	1.00000	0.50000
10	Te	0.50000	0.50000	1.00000

Te3 group

8	Te	0.50000	-0.50000	1.00000
9	Te	1.00000	0.50000	-0.50000
10	Te	-0.50000	1.00000	0.50000

Cl1 group

11	Cl	1.00000	1.00000	1.00000
12	Cl	-0.33333	0.33333	0.33333
13	Cl	0.33333	-0.33333	0.33333
14	Cl	0.33333	0.33333	-0.33333

Cl2 group

12	Cl	1.00000	0.50000	0.50000
13	Cl	0.50000	1.00000	0.50000
14	Cl	0.50000	0.50000	1.00000

Cl3 group

12	Cl	0.50000	-0.50000	1.00000
13	Cl	1.00000	0.50000	-0.50000
14	Cl	-0.50000	1.00000	0.50000
